# ‘Recoupling’ the attentional and motor control of preparatory postural adjustments to overcome freezing of gait in Parkinson’s

**DOI:** 10.1186/s12984-020-00776-1

**Published:** 2020-10-31

**Authors:** Amy Maslivec, Anna Fielding, Mark Wilson, Meriel Norris, William Young

**Affiliations:** 1 Department of Surgery and Cancer, Imperial College London, London, UK; 2 College of Health and Life Sciences, Brunel University London, Uxbridge, UK; 3 School of Sport and Health Sciences, University of Exeter, Exeter, EX1 2LU UK

**Keywords:** Parkinson’s, Freezing of gait, Cueing, Step initiation, Anticipatory postural adjustment, Weight-shifting, Festination, Start hesitation

## Abstract

**Objectives:**

This study examined if people with Parkinson’s and freezing of gait pathology (FoG) could be trained to increase preparatory weight-shift amplitude, and facilitate step initiation during FoG.

**Methods:**

Thirty-five people with Parkinson’s and FoG attempted to initiate forward walking from a stationary position caused by a freeze (n = 17, FoG-F) or voluntarily stop (n = 18, FoG-NF) in a Baseline condition and two conditions where an increased weight-shift amplitude was trained via: (i) explicit verbal instruction, and (ii) implicit movement analogies.

**Results:**

At *Baseline*, weight-shift amplitudes were smaller during: (i) unsuccessful, compared to successful step initiations (FoG-F group), and (ii) successful step initiations in the FoG-F group compared to FoG-NF. Both *Verbal* and *Analogy* training resulted in significant increases in weight-shift amplitude in both groups, and a corresponding pronounced reduction in unsuccessful attempts to initiate stepping (FoG-F group).

**Conclusions:**

Hypometric preparatory weight-shifting is associated with failure to initiate forward stepping in people with Parkinson’s and FoG. However, impaired weight-shift characteristics are modifiable through conscious strategies. This current study provides a novel and critical evaluation of preparatory weight-shift amplitudes during FoG events. The intervention described represents an attractive ‘rescue’ strategy and should be further scrutinised regarding limitations posed by physical and cognitive deficits.

## Highlights

- Unsuccessful attempts to initiate a step from a freeze is associated with hypometric medio-lateral weight-shifting
- People with Parkinson’s can voluntarily increase the amplitude of medio-lateral weight-shift
- Voluntarily increasing medio-lateral weight-shift is associated with successful forward step initiation

## Introduction

Freezing of Gait (FoG)—defined as a sudden inability to initiate or continue walking—affects more than 50% of people with Parkinson’s disease (PD) [1]. FoG is recognised as one of the most debilitating symptoms of advanced Parkinson’s and is associated with injurious falls [2], anxiety and depression, and reduced quality of life [3]. Current management of FoG includes the attempted optimisation of pharmacological and surgical interventions. However, the efficacy of current approaches is limited [4], leading to an increasing emphasis on developing new approaches to physical therapies [5] in conjunction with options for managing anxiety [6] and cognitive decline [7].

The pathophysiology of FoG, although still controversial, has been attributed to five main mechanisms [8]. Abnormal coupling between posture and gait (e.g. impaired weight shifting in preparation to initiate walking) is one of those. While much research has focussed on factors that contribute to freezing onset (i.e. the cessation of gait) [4, 9], the current study describes an intervention aimed at facilitating weight-shifting and step initiation from FoG, while considering the effects of both anxiety and factors that limit cognitive resources when performing motor skills.

The transition between an upright static posture and walking is primarily characterized by a shift of the centre of pressure laterally toward the stance leg, which serves to unload the stepping leg, allowing it to swing as the centre of mass is propelled forward. This weight shift constitutes the latter phase of the so-called anticipatory postural adjustment (APA) [10], otherwise referred to as the ‘unloading phase’ [11]. The necessity to sufficiently unload the stepping limb has motivated attempts to better characterise APAs in people with PD, particularly in those with FoG pathology.

Early reports of Parkinson’s–related hypometric APAs during self-initiated gait [12, 13] led to claims that defective APAs might represent a major underlying mechanism (and cause) of FoG [8]. However, a recent report has created uncertainty in this debate. Schlenstedt et al. [14] showed that lateral APA weight shifts were indeed impoverished in Parkinson’s patients with FoG compared to those without FoG. However, like the majority of extant literature, these observations only relate to circumstances where gait initiation was successful. In a number of trials containing start hesitations (too few for statistical analysis), FoG was associated with larger APA amplitudes. Furthermore, APA magnitude during successfully initiated steps was proportionate to self-reported FoG severity; findings that led the authors to suggest that increased APA magnitude may represent a compensatory mechanism intended to reduce destabilising accelerations in the centre of mass [14]. Indeed, descriptions of reduced FoG-related weight-shifting and a variety of compensatory strategies appear in anecdotal reports from patients and clinicians [15]. However, current literature describing the relationship between APA scaling and gait initiation success during/immediately preceding a freeze is limited and somewhat conflicted.

It is notoriously difficult to induce and evaluate FoG in laboratory settings, as illustrated by the scarcity of direct within- and between-subject comparisons in the literature. To our knowledge, no study has consistently induced FoG in a manner sufficient to afford robust comparisons of APA magnitude (of the unloading phase) between successful and unsuccessful attempts to initiate stepping from a freeze. This was the first aim of the current study.

### Training APAs as an intervention

FoG is generally considered to be heterogeneous and several conceptual models have emerged that attempt to explain the underlying pathophysiology. The prominent ‘decoupling theory’, proposed by Lewis and Barker [16], suggests that FoG is a manifestation of a dissociation between a pre-planned motor program and motor initiation, leading to compromised motor output. The regulation of these processes is thought to rely to some extent on attentional and executive resources that are often deficient in people with Parkinson’s and FoG pathology. Indeed, repeated observations that external factors can both exacerbate and alleviate FoG provide a clear indication of the influence that altered attentional processes can exert on FoG [5].

Tard et al. [17] demonstrated FoG-related differences in the way attention is allocated during step preparation. More specifically, they claimed that post-perceptual, high-level attentional processes are responsible for the (potentially premature) release of defective APA motor programs. Their inherent assertion is that effective suppression of preparatory motor commands relies on attentional and executive resources that are often deficient in patients with FoG [3, 18–20]. Indeed, increasing attentional demands by means of a dual-task paradigm leads to reduced medio-lateral weight shifting during step preparations in patients with FoG [17, 21]. This ineffective suppression of preparatory motor commands is also exemplified in the contextualisation that knee trembling represents sequential/excessive APAs [22].

Many studies attempting to manipulate attention in the context of FoG have used dual-task paradigms where attention is, at least in part, allocated to task-irrelevant processes [23, 24]. The consequential decline in stepping performance supports the notion that interference between neural circuits might be suspended if patients with FoG allocate attention towards task-relevant—and more specifically; goal-directed—behaviour [16, 18]. This presents an opportunity for the development of novel therapeutic strategies that incorporate the production of an APA (particularly on the critical unloading phase) into the intended movement goal [25, 26]. In conjunction with previous suggestions, we anticipate that this process will de-automatize the basic defective APA, thereby facilitating motor output [27]. As such, the second (and primary) aim of the current study was to evaluate the above prediction. We aimed to generate sufficient data to document the amplitude of medio-lateral weight-shifting (ML-WS) and the proportion of successful attempts to initiate stepping from a freeze, and to compare these outcomes between a Baseline and two experimental conditions denoting specific changes in goal-directed attentional focus.

### Verbal control of weight-shifting

In early stages of learning/re-learning motor skills, performers will typically rely on explicit verbal rules that specify the most fundamental characteristics of the task [28]. However, such processes can become detrimental in later stages of learning through the disruption of movement automaticity (i.e., breaking down the automated skill into smaller steps and increasing the chance of producing errors, [29]). This over-reliance on verbal movement rules, also known as ‘reinvestment’ [30], is associated with increased cognitive task demands [31] and a decline in motor performance during gait in healthy young [32] and older adults [33], and in a range of sporting, balance, and vocational tasks [34, 35].

Over-reliance on conscious movement processing is often observed when people experience performance anxiety [30], rendering this issue particularly relevant for people with PD, especially those with FoG [36]. Previous work had already shown that the number of years since diagnosis is associated with self-reported ‘reinvestment’ in Parkinson’s [37]; a potential product of both: (i) PD-related deficits in movement automaticity, and, (ii) anxiety/concern about movement, be it related to physical safety, social concerns and/or self-appraisal [38]. Based on this potential preference and/or need to use conscious movement processing, the current study utilised simple verbal instructions related to APA production. However, given the evidence linking such processes with both cognitive and motor inefficiencies during gait tasks [39], there is also a need to explore alternative options for training/cueing APAs that might promote implicit movement control and overcome these inefficiencies.

### Using analogies to facilitate APAs

One potential approach—analogy learning—a technique initially developed for learning sporting skills that are subsequently robust under performance anxiety [30], has recently shown encouraging results in the field of neurorehabilitation [40, 41]. Analogies involve repackaging relevant explicit (verbal) information of the to-be-learned skill into one integrated biomechanical analogy or metaphor. Analogies strive to combine information into chunks, and a meaningful analogy should lead individuals to retrieve larger chunks, therefore utilising relatively efficient unconscious processes [42, 43]. People who use implicit cues (such as analogies) are less likely to excessively try to control their movements and therefore maintain more robust motor performance in anxious situations [29]. A recent study has successfully employed the analogy of ‘following footprints on a sandy beach’ to improve gait characteristics in PD without FoG [44]. Previous work has also shown that PD with FoG can step in place for longer durations before freezing when using auditory cues designed to exploit similar implicit mechanisms [45].

In the current study we therefore included an experimental condition pertaining to APA-related analogies. We predicted that both *Verbal* and *Analogy* manipulations of APA characteristics would lead to significant increases in ML-WS amplitude and associated improvement in successful step initiation from a freeze. However, we anticipated that observed improvements in stepping performance would be more pronounced when using movement analogies, due to associated efficiencies in cognitive processing.

## Methods

### Participants

Thirty-five participants diagnosed with Parkinson's were recruited through Parkinson’s UK advertisements and local Parkinson’s UK branches. All participants experienced regular freezing of gait (responding with a score of 3 or greater to the third item in the Freezing of Gait Questionnaire; [46]). Participants were excluded from the study if they had cognitive deficits (MiniCog score of < 3; [47]), or reported any musculoskeletal or neurological issue (other than Parkinson's) that significantly affected their walking. Prior to testing, all participants self-reported that they were able to stand unsupported for at least 60 s. While all participants typically experienced FoG at least once a day, they were divided into two groups based on whether they exhibited at least three recorded freezes within each condition described below (FoG-F, n = 17). For those participants exhibiting fewer (n = 3) or no (n = 15) freezes (FoG-NF, n = 18), analyses were carried out on successful steps from a voluntary stop. For the FoG-F group, data from the Baseline condition were entered into an initial comparison between successful and unsuccessful steps from a freeze. When comparing outcomes between experimental conditions, only data concerning successfully initiated steps were included. This approach was determined a priori*,* to afford direct comparisons to the FoG-NF group. Furthermore, such statistical comparisons were not feasible due to an insufficient number of unsuccessful attempted steps occurring during Verbal and Analogy trials.

The Unified Parkinson’s Disease Rating Scale motor section (UPDRS-III) was administered by a certified researcher (see Table 1).

**Table 1:** Participant characteristics

		Non freeze (n = 18)	Freeze (n = 17)	*P*
Age		66.5 ± 9.24	69.9 ± 9.9	.313
Sex (M/F)		10/8	10/7	.542
Years since diagnosis		9.6 ± 7.5	8.6 ± 6.1	.685
MoCA (0–30)		26.8 ± 4.8	27.1 ± 1.8	.266
FES (0–100)		65.8 ± 19	62.1 ± 19	.654
FOGQ (0–24)		15.1 ± 2.9	16.3 ± 4.7	.337
UPDRS III (0–108)		19.13 ± 3.7	22.9 ± 5.3	.062
H&Y stage (1–5)		2.3 ± 0.7	2.8 ± 0.9	.123

### Experimental design

The testing session was carried out between one and two hours after participants received their usual dopaminergic medication (i.e., the ‘ON’ state). All testing was completed within a single session. Participants were instructed to step in place (SIP) (i.e., alternately lift each foot from the ground without progressing in any direction) on a single force plate (dimensions = 600 × 400 mm; Kistler Group, Switzerland) and to keep stepping for 60 s or until a freeze occurred that resulted in the cessation of stepping. Participants were given the prior instruction (before the start of each trial) that, if a freeze occurred, they should attempt to initiate forward walking as they usually would using either foot. If no freeze occurred that led to a cessation of stepping within the 60 s trial, after 60 s of stepping, participants were asked to “stop”. They then attempted to start walking forwards as per their original instructions. With the exception of the latter (concerning only FoG-NF participants), verbal instructions were confined to the period before each trial. At the start of the session, participants were instructed to practice stopping the SIP task and initiating forward walking at least three times in order to familiarise themselves with the protocol. Stepping trials were repeated up to six times (or until four attempted steps from FoG events were recorded) for each of three different conditions; Baseline, Verbal and Analogy.

Participants were securely fitted into a safety harness for all stepping trials and were given the opportunity to stop, sit down and have refreshments at any point between trials. Each stepping trial was completed once the participant had made an attempted step forward (successful or otherwise). At the end of each trial, participants were asked to return to their original position on the force plate (if necessary) with the help of a researcher to begin the next trial.

Inducing FoG in laboratory settings represents a significant challenge. Therefore, we integrated two procedures that have previously been shown to expedite FoG. First, the SIP task has been repeatedly shown to successfully induce FoG [45, 48]. This task also carries the additional benefit that participants’ movements can be constrained so that, if FoG occurs, participants will be positioned on a force plate, thus permitting the necessary recordings. If the SIP task did not successfully induce FoG, participants were invited to suggest alternative strategies. Consequently, three participants in the FoG-F group experienced—and attempted to initiate forward gait from—freezes induced by a combination of SIP and turning from on the spot. Here, only trials were used when the freeze resulted in participants’ feet both facing in the same direction as the intended walking path (to maintain task consistency between participants).

Second, participants wore a virtual reality head-mounted display (HTC Vive, Sony Ltd) displaying one of two environments designed in Unity3D (Unity Technologies) to induce freezing by presenting scenes commonly observed to exacerbate FoG [8] and, similarly, through elevating anxiety [6]. These environments depicted participants standing either at the top of a set of descending stairs with no handrail or in front of an open narrow doorway with tables and chairs on the other side. Participants were asked to select an environment to use throughout the study, based on which, they felt, was most likely to induce FoG. The visual scene was updated based on participants’ head movements and forward progression during successful steps. The nearest constraints in the virtual environments were placed approximately 2 m away from participants’, meaning that they did not ‘contact’ or interact with them (e.g. walk through the door or down the stairs). Prior to stepping trials, participants were given time to familiarise themselves with the VR environments in both seated and standing positions. Ten participants (4 FoG-F and 6 FoG-NF) selected to complete all stepping tasks without using the VR environment on the premise that they would be more likely to freeze without the headset on (as opposed to any reports of motion sickness or feelings of discomfort).

In the Baseline condition, prior to the start of each trial participants were given the instruction that when they stopped stepping (be it following a freeze or voluntary stop), that they should start walking forwards in their preferred manner (the use of physical devices to assist gait initiation was prohibited).

**Fig. 1:**
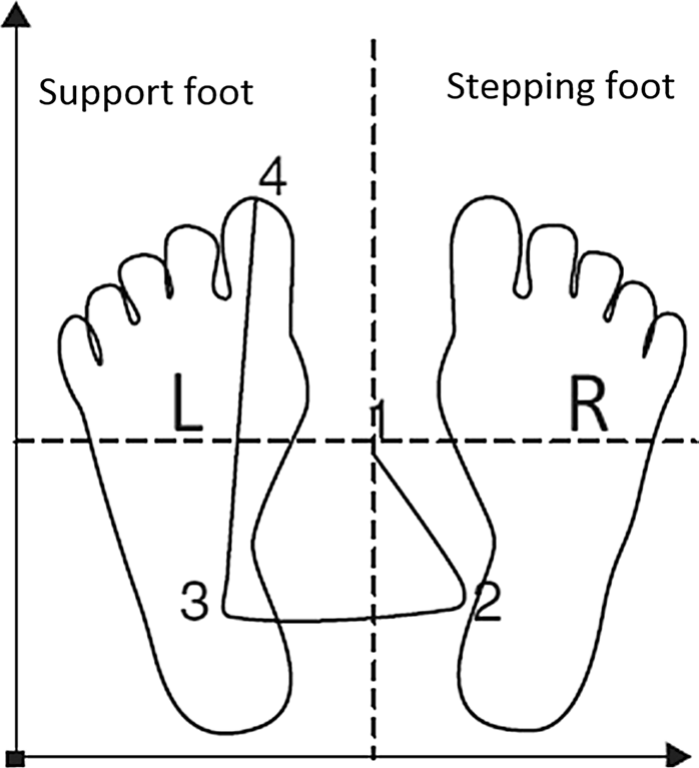
Image exemplifying an APA shown to participants during verbal and analogy training

In experimental conditions, participants were trained to use a weight shifting strategy based on a simplified description of an APA. During training, participants were invited to practice “Moving [their] weight sideways slightly towards the stepping leg, then swaying back and shifting all [their] weight on to the non-stepping leg before stepping forward”. Demonstrations were given by the researcher during training and participants were shown a printed image exemplifying an APA for reference (Fig. 1). Participants practiced the movement up to ten times, and in doing so, were asked to concurrently verbalise the instructions to move “right, left and forwards” (for those initiating gait with their right foot) or as a number sequence “One, two and three”. This allowed the researcher to confirm that participants understood the instructions and to give participants the opportunity to ask any questions about the Verbal strategy. During the Verbal condition, when participants stopped stepping (due to a freeze or voluntary stop) they were given the prior instruction to use the newly-learned verbal strategy to initiate forward walking.

Participants were also trained to use a weight shifting strategy equivalent to the Verbal strategy, but in the form of a movement analogy. Participants were shown a printed image exemplifying an APA (same as Verbal condition) and the researcher gave both a description and demonstrations of the required weight-shifting movement, being careful not to give simplified verbal cues. The researcher then explained the concept of using movement analogies as a strategy to produce the required weight shift and initial forward step. Participants were given some examples of analogies to practice moving to. For example, “imagine you are stood on a set of traditional balancing scales, when one foot presses down, the other foot lifts up and makes a step”. Other examples related to swaying like a tree in the breeze or like a slalom skier. Participants were also encouraged to create their own personalised analogies in an attempt to make the process more meaningful and memorable to the individual (e.g., [49]). Thirteen participants opted to create and use their own analogies (FoG-F: n = 7/17, FoG-NF: n = 6/18). These included shifting weight like: (i) a rugby player performing a ‘dummy’ turn, (ii) a tennis player waiting to receive a serve, (iii) a boxer moving towards an opponent, and (iv) a person standing on a moving boat. During the analogy trials, when a freezing episode occurred or the researcher instructed the participants to stop, participants thought of their chosen analogy to perform the weight shifting movement to make a step forward.

Participants practiced shifting weight/initiating a step using their chosen analogy up to ten times. During the Analogy condition, when participants stopped stepping (due to a freeze or voluntary stop) they attempted to use the newly-learned Analogy strategy to initiate forward walking, as per instructions given prior to the start of each trial.

The order of Verbal and Analogy conditions was counterbalanced. However, participants always completed the Baseline condition first in order to avoid carry-over effects (i.e., participants using any strategies learned in the other conditions during Baseline trials). Immediately following the final trial in each condition, participants were asked to retrospectively describe their thoughts when attempting to initiate forward walking in preceding trials. Responses were audio recorded, transcribed verbatim and allocated into one of four categories: (i) Focus on environment, future planning or global aims (e.g., “thinking about doorway/stairs”, “counting how many steps to reach the doorway”, “just move”); (ii) Instructions specific to foot movement (e.g., “pick your foot up”, “step forward and [put] heel down first”); (iii) Instructions specific to weight-shifting (e.g., “counting 1, 2, 3 [to shift weight as instructed]”, “move right, left, then forward”); and (iv) Analogies relevant to weight-shifting. We counted the number of participants reporting thought processes in each category. These data were not intended for any statistical analysis, but rather as a broad evaluation of the fidelity of each condition/manipulation.

A nine camera motion analysis system (VICON, Oxford Metrics, London, England) was used to reconstruct the position of reflective markers on the lateral malleolus, calcaneus and the head of the fifth metatarsal of each foot at a sampling rate of 100 Hz, and filtered with a fourth order Butterworth low pass filter with a cut-off frequency of 5 Hz. A single force plate was used to record medio-lateral (ML) centre of pressure (COP) motion with a sampling frequency of 1000 Hz. All analyses involving signal processing were carried out using bespoke scripts in Matlab (Mathworks Inc.), where the researcher was blinded to the condition pertaining to each trial. In all cases, data were plotted and visually checked to avoid artefacts.

### Data analysis

A freezing of gait episode was defined both subjectively and objectively. Stepping trials were visually evaluated through playback of steps recorded on a video camera and outputs from the motion capture system. Audio recordings from this video were also used to document participants’ verbal reports of any freeze that they perceived. A FoG episode was defined as an episode of involuntary cessation of gait which was often accompanied by trembling of the legs, of festination, and when the vertical and anterior displacement of the toe marker dropped between zero and one standard deviation of the initial value of that trial [50]. If FoG was subsequently confirmed through this initial process, motion capture and force plate data relating to each step during the trial were exported and analysed.

**Fig. 2:**
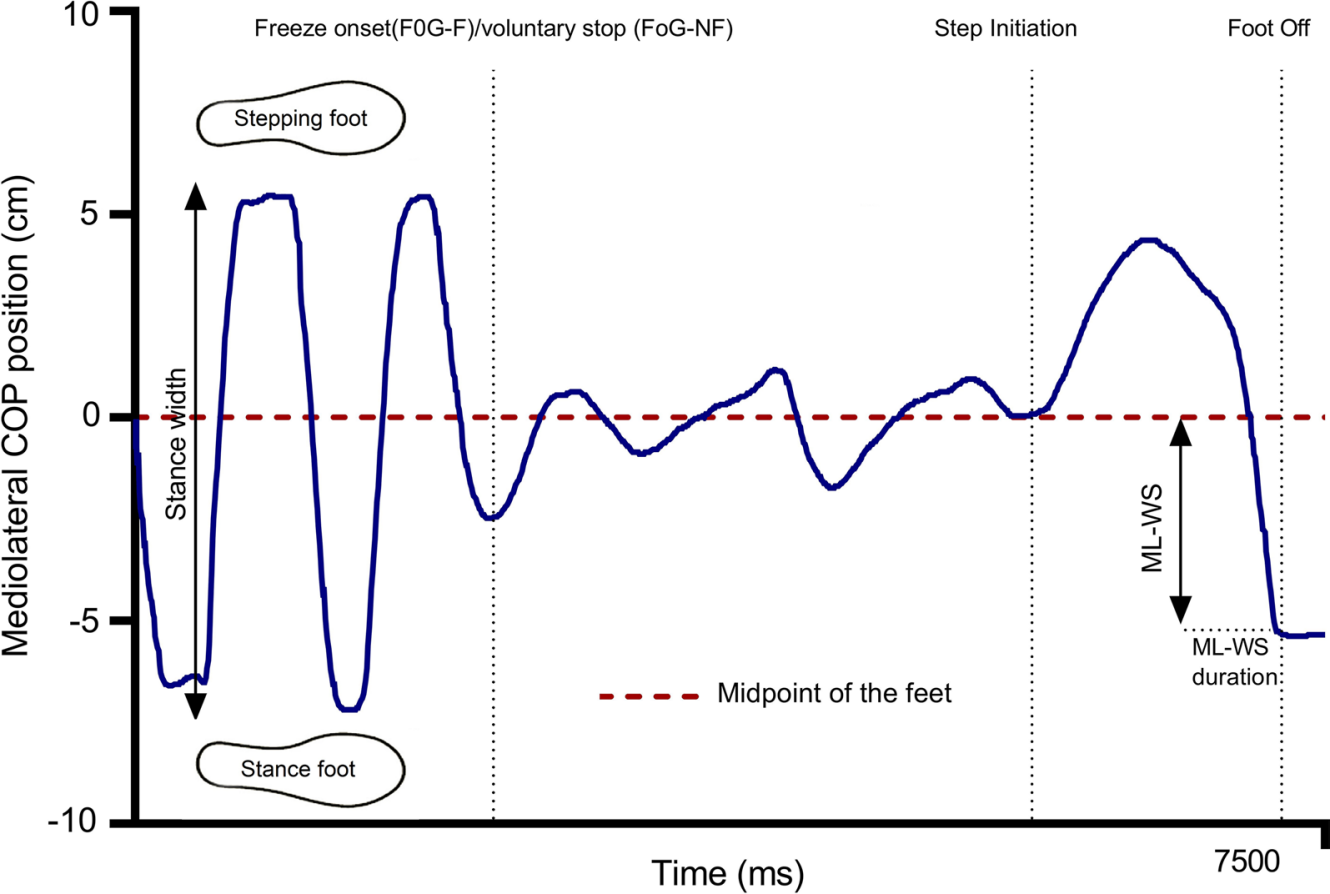
Exemplified calculation of ML-WS

The onset of an APA (first measurable change in ML COP from freezing episode or stopped position) was detected by an automated threshold-based algorithm, with the threshold set at three standard deviations of the mean ML COP displacement of the freezing episode/stopped position. The mean and standard deviation were taken from the maximum time available up to 1000 ms following to the freeze or stop. A total of eight trials across four participants contained a period of 400–1000 ms. ML-WS amplitude was assessed by analysing the peak ML displacement of the COP after it was displaced from the midpoint between the heel markers on each foot to the maximum lateral shift towards the stance foot before forwards propulsion was evident. Forwards propulsion was detected using a threshold at two standard deviations of the mean AP COP displacement during the lateral shift to the stance foot (Fig. 2). Resultant values were then normalised to stance width by calculating the percentage of stance width divided by 2. Our metric of ML-WS therefore describes the lateral weight-shift achieved only during the preparatory lateral unloading phase of the APA (prior to forward propulsion). ML-WS duration was the time of ML-WS. Stance width was calculated as the distance between heel markers on each foot during the freeze/stopped position prior to attempted stepping.

For trials where participants verbally reported having experienced a freeze, we evaluated whether subsequent attempts to initiate forward walking from that freeze was successful or not (FoG-F group only). Video recordings of each trial were viewed and rated by three independent evaluators who were blinded to each condition. Each evaluator made categorical judgements about whether the participant, in their initial attempt, appeared to successfully or unsuccessfully initiate a forward step [51]. An unsuccessful step was identified as a start hesitation/failed attempt to step from a freeze and was determined when a participant displayed one or more heel-off movement(s) without moving forward. This was then confirmed using the vertical displacement of the heel marker on the motion capture system. Any discrepancy between evaluators resulted in a communal viewing of the relevant trial, a discussion regarding the rationale for the decisions made and a consensus reached. Throughout the entire dataset, this process was only necessary for 1 trial. Again, evaluators remained blinded to the trial condition throughout this process. We calculated the mean ratio of successful to unsuccessful attempts.

Failed attempts to initiate forward walking from a freeze (as identified by the independent evaluators) were timestamped on the video recordings and corresponding motion capture and force plate data for that time period were exported and analysed. To determine ML-WS for these unsuccessful attempts to step, we calculated the ML displacement of the COP from the midpoint of the feet to the maximum lateral shift towards the stance foot during the period immediately preceding the observed start hesitation. ML-WS was then normalised to stance width (as described above). Where participants made multiple APA’s in an attempt to make a successful step, only the first APA was used to calculate ML-WS. Only during unsuccessful steps is it uncertain whether peak lateral COP position reached the location of the stance limb (i.e. 100% of stance width as calculated above). We therefore calculated both ML-WS and the global peak lateral COP position (normalised to stance width as above) during unsuccessful attempted steps. This was to determine if any significant additional lateral weight-shift occurred following the preparatory unloading phase/onset of forward propulsion (if present). For two participants in the FoG-F, it was not possible to reliably determine the point of attempted step initiation in any trials. Therefore, comparisons of ML-WS between successful and unsuccessful attempted steps were carried out on data from the remaining 15 FoG-F participants.

### Statistical analysis

We conducted a paired-samples t-test to compare ML-WS and global peak lateral COP position between successful and unsuccessful attempted steps in FoG-F during Baseline trials. Effects sizes are expressed as Cohen’s D. We also carried out Spearman’s Rank Order correlations to evaluate possible associations between ML-WS amplitude when stepping at Baseline (where participants were not attempting to directly manipulate APA characteristics), disease severity (UPDRS III and H&Y Stage) and self-reports of freezing severity (FOGQ).

A mixed design ANOVA (2 × 3 design) was performed for ML-WS, ML-WS duration and stance width, with group (FoG-F and FoG-NF) as a between-subject factor and stepping conditions (Baseline, Verbal and Analogy) as a within-subject factor. Significant (*p* < 0.05) effects were followed-up with Holm-Bonferroni-corrected post-hoc tests and effects sizes are expressed as partial eta^2^ (ANOVA) or Cohen’s d (t-test). Wilcoxon signed-rank tests were used to compare the ratio of successful to unsuccessful steps between conditions in the FoG-F group and effect sizes are expressed as $r\left( {\frac{z}{{\surd {\text{N}}}}} \right)$.

## Results

### Factors associated with altered medio-lateral weight-shift amplitude

**Fig. 3:**
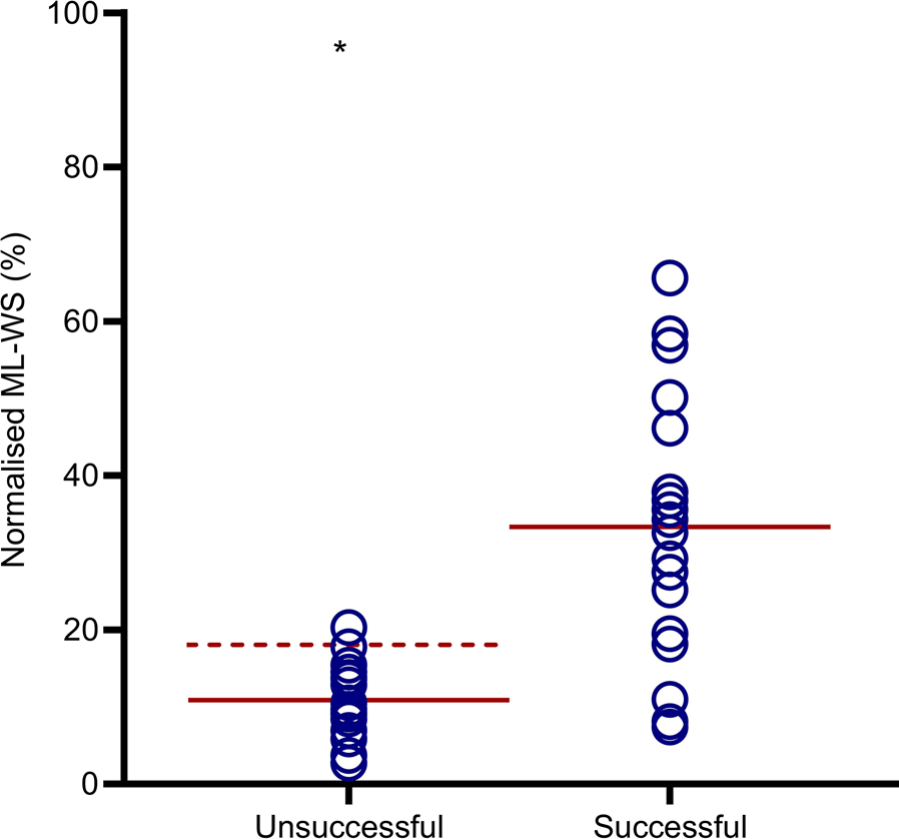
Mean normalised ML-WS in the FoG-F group is smaller during unsuccessful compared to successful attempts to initiate forward stepping. **p < 0.001. Solid horizontal lines indicate mean ML-WS. Dashed horizontal line indicated peak lateral displacement of COP

Results from the FoG-F group showed that, when comparing successful to unsuccessful attempts to initiate forwards stepping from a freeze, normalised ML-WS amplitude was significantly lower preceding unsuccessful attempts (t_(15)_ = − 5.186, *p* < 0.001, *d* = 1.08). Normalised global peak of lateral COP displacement of unsuccessful steps was also significantly lower than normalised ML-WS during successful steps (19.11 ± 12.1 vs 34.12 ± 19.5% (t_(15)_ = − 4.150, *p* < 0.002, *d* = 0.86)) (see Fig. 3).

**Fig. 4:**
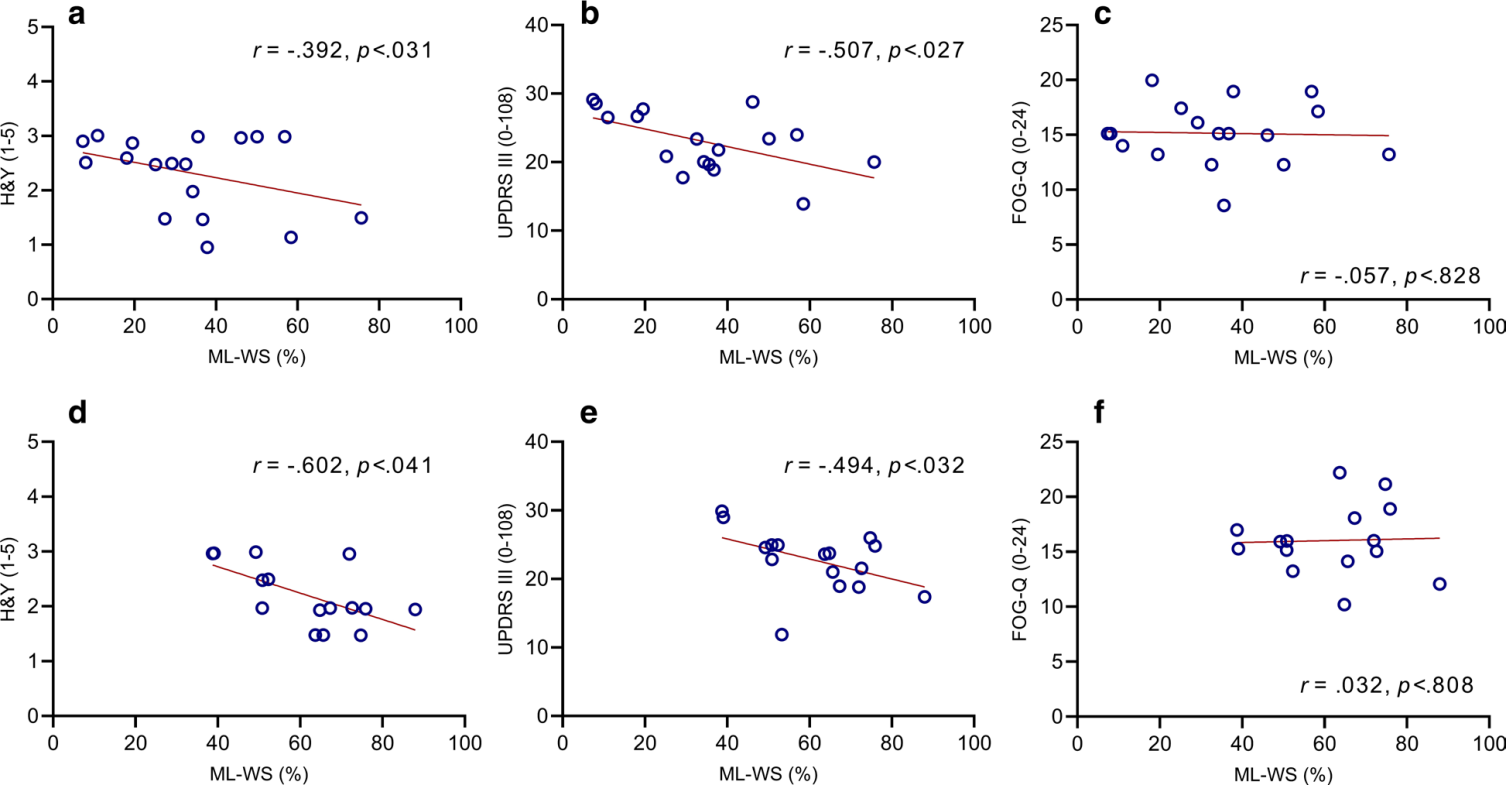
Correlations between normalised ML-WS (%) and **a** H&Y, **b** UPDRS III, and **c** FOGQ score in the FoG-F (top) and FoG-NF group (bottom)

Correlation analyses carried out on successful steps made in both participant groups revealed that, regardless of whether participants were attempting to step from a freeze (FoG-F) or a voluntary stop (FoG-NF), significant moderate negative associations were observed between ML-WS and both H&Y and UPDRS-III motor examination scores. However, no association was observed with self-reported FoG severity (see Fig. 4).

### Effects of verbal and analogy training

#### ML-WS amplitude

**Fig. 5:**
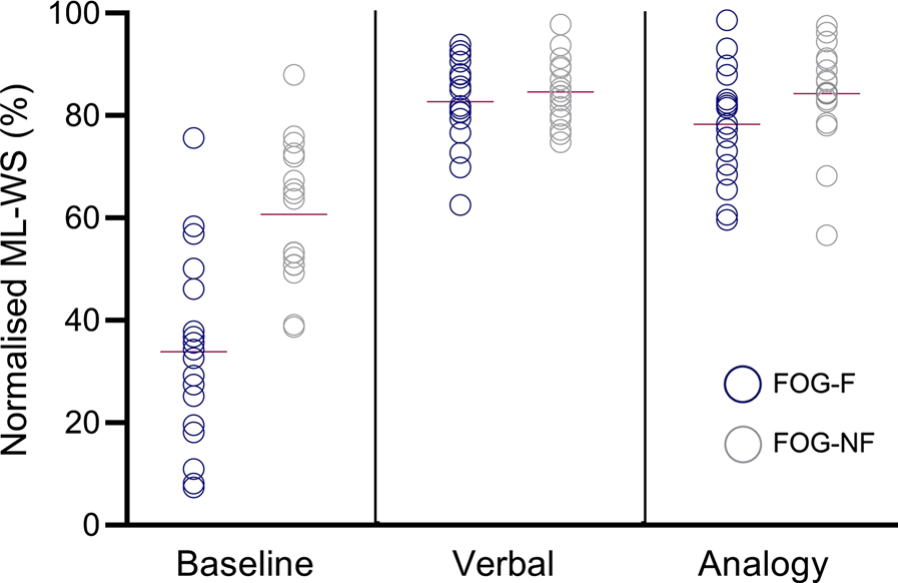
Normalised ML-WS (%) significantly increased in Verbal and Analogy conditions compared to Baseline in both FoG-F and FoG-NF groups

The results of the Two-Way ANOVA showed a significant main effect of condition on normalised ML-WS, F_(2,66)_ = 134.477, *p* < 0.001, n^2^ = 0.803, and a significant interaction between Group and Condition, F_(2,66)_ = 14.807, *p* < 0.001, n^2^ = 0.310. There was no significant group effect F_(1,33)_ = 1.984, *p* = 0.187, n^2^ = 0.068. Within-subject comparisons showed that both FoG-F and FoG-NF participants exhibited significantly lower ML-WS amplitude at Baseline compared to Verbal, and Analogy conditions (FoG-F, Verbal(t_(16)_ = − 10.565 *p* < 0.001, *d* = 1.124) and Analogy (t_(16)_ = − 11.623, *p* < 0.001, *d* = 1.22)) and (FoG-NF, Verbal(t_(17)_ = -7.823 *p* < 0.001, *d* = 1.04) and Analogy (t_(17)_ = − 6.657, *p* < 0.001, *d* = 0.98)). Between-group comparisons showed that FoG-F participants exhibited significantly lower ML-WS amplitude at baseline compared to FoG-NF (t_(33)_ = 4.85, *p* < 0.001, d = 1.35). However, no significant differences were evident between groups for Verbal (t_(33)_ = − 0.514, *p* < 0.611, d = 0.016) and Analogy conditions (t_(33)_ = 0.169, *p* = 0.198, d = 0.12) (Fig. 5).

#### ML-WS duration

Results showed a significant main effect of condition on ML-WS duration, F_(2,66)_ = 25.021, *p* < 0.001, n^2^ = 0.426, but no significant interaction, F_(2,66)_ = 2.535, *p* = 0.087. There was no significant group effect of condition F_(1,33)_ = 1.54, *p* = 0.698, n^2^ = 0.005. Participants exhibited shorter ML-WS duration at baseline compared to Verbal (t_(33)_ = − 4.990, *p* < 0.05, *d* = 0.64) and Analogy (t_(33)_ = − 6.564, *p* < 0.05, *d* = 0.81) conditions.

#### Stance width

There was no main effect of condition on stance width, F_(2,66)_ = 1.547, *p* = 0.220, n^2^ = 0.045, and no interaction effect, F_(2,66)_ = 0.835, *p* = 0.438, n^2^ = 0.025. There was no significant group effect of condition, F_(1,33)_ = 1.28, *p* = 0.723, n^2^ = 0.004. (Fig. 5).

#### Ratio of successful to unsuccessful attempts to step from a freeze

**Fig. 6:**
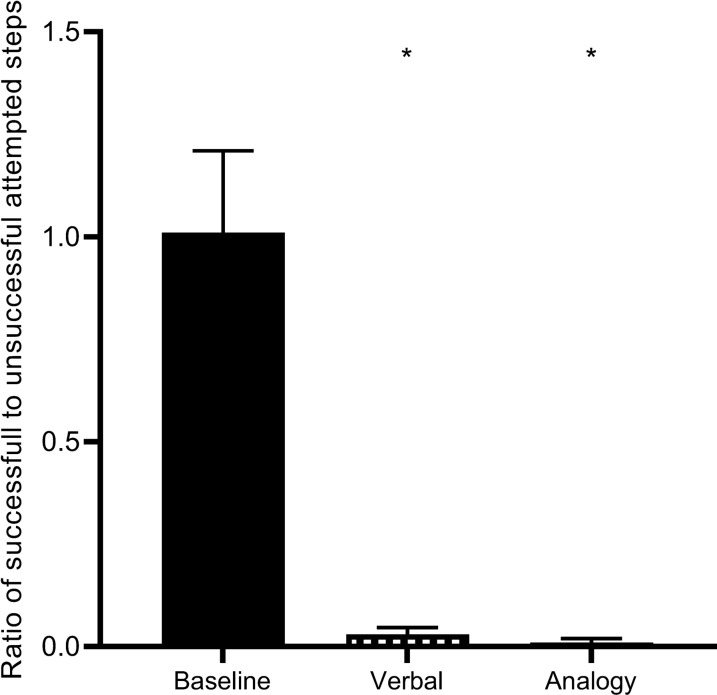
The ratio of successful to unsuccessful attempts to step from a freeze (in the FoG-F group) reduces in Verbal and Analogy conditions compared to Baseline

Our results show that FoG-F decreased the proportion of unsuccessful attempts to initiate a step from a freeze between Verbal to Baseline conditions (z = − 3.301, *p* < 0.001, *r* = 0.82) and Analogy to Baseline conditions (z = − 3.301, *p* < 0.001, *r* = 0.82). There was no difference in the proportion of unsuccessful steps between Verbal and Analogy conditions (z = − 1.069, *p* = 0.285, *r* = 0.4). During baseline trials, FoG-F made 69 unsuccessful and 73 successful attempts to step from a freeze, resulting in an overall ratio of 1:1.01 (successful: unsuccessful). During *Verbal* and *Analogy* conditions, FoG-F made 72 and 79 successful, and only 3 and 1 unsuccessful steps, resulting in an overall ratio of 1:0.03 and 1:0.01, respectively (Fig. 6).

#### Self-reported thoughts during attempted forward step-initiations

The number of participants reporting thought processes from each pre-defined category are described in Table 2 (pooled data from both groups). These results indicate that participants typically utilised verbal instructions relevant to initiating foot movement during Baseline trials. During *Verbal* trials, all participants self-reported thinking about the instructions provided. While this was also largely the case in *Analogy* trials, almost two-thirds of participants also self-reported using verbal instructions about weight-shifting in addition to relevant analogies.

**Table 2:** The number of participants reporting thought processes from each category during baseline, verbal and analogy

Category of thought processes				
Condition	Focus on environment or future Planning	Instructions specific to foot movement	Instructions specific to weight-shifting	Analogies relevant to weight shifting
Baseline	26 (74%)	26 (74%)	1 (2.8%)	0 (0%)
Verbal	5 (14.3%)	10 (28.5%)	35 (100%)	0 (0%)
Analogy	4 (11.4%)	7 (20%)	22 (62.9%)	34 (97.1%)

Values represent pooled data from FoG-NF and FoG-F groups

## Discussion

### Factors associated with weight-shift amplitude at Baseline

To our knowledge, this is the first instance where sufficient data has been generated to afford statistical comparisons of APA characteristics between: (1) successful and unsuccessful attempts to step from a freeze (within-subject) and ii) attempts to initiate gait from both a freeze (FoG-F) and a voluntary stop (FoG-NF). We attribute this success in inducing FoG in our laboratory setting to the use of the SIP task in conjunction with the VR environments designed specifically to represent scenarios commonly associated with exacerbated FoG events (e.g., narrow doorways). As such, while we acknowledge limitations associated with using VR head-mounted displays and the necessary contrived laboratory environment/task, we also suggest that our current data may, in comparison to extant literature, better reflect behaviour of patients attempting to initiate gait in daily life.

Previous work has documented impoverished APAs in PD with FoG when initiating gait in the absence of any observed freeze [13, 26], leading to suggestions that FoG may be a consequence of attenuated/defective APA scaling [8]. Our findings provide novel and additional support for this notion, as ML-WS amplitudes were significantly attenuated during attempted steps that were unsuccessful (Fig. 3).

Schlenstedt et al. [14] compared the magnitude of ML APAs between groups of PD patients that were categorised according to presence/absence of self-reported FoG pathology. They reported no significant between-group differences at baseline during successfully initiated steps; findings that conflict with ‘traditional’ conceptualisations described above. Their study also provided data relating to nine attempted steps where start hesitations were observed and determined that APA amplitude was relatively large compared to step initiations where FoG was absent. The authors interpreted these trends as representing a potential compensatory mechanism aimed at avoiding destabilising accelerations in the centre of mass. These interpretations were supported by positive associations observed between self-reported FoG and APA amplitude [14] and velocity [52]. Our data indicate that no such relationship exists, at least not concerning our measure of ML-WS (Fig. 4 C&E); a finding that is consistent across participant groups (i.e., it is not contingent on whether individuals are attempting to step from a freeze or voluntary stop). However, despite this apparent discrepancy, we argue for a similar interpretation and conclusion. Our findings from the FoG-F group at Baseline show that smaller ML-WS amplitudes are associated with unsuccessful attempts to step. Therefore, occasions where participants increased ML-WS (and successfully initiated steps from a freeze) might indeed represent an effective compensatory strategy to overcome FoG, as suggested by Schlenstedt et al. [13]. The question remains whether users must be cognisant of processes involved in the production of larger weight-shifts in order to attain improvements in motor performance.

We must be cautious when contextualising the evident relationship between increased ML-WS and the improved rate of successful gait initiations (Fig. 6). Specifically, it is unclear if the hypokinetic ML-WS described during unsuccessful attempts to initiate walking (Fig. 3) should be considered as a mechanism primarily responsible for resultant failed attempts, or as one of several integrated behaviours that are symptomatic of more fundamental defective mechanisms (i.e., an artefact of the failed step). Similarly, the production of larger ML-WS during experimental conditions may not represent the most important factor driving improvements in step initiation. For example, benefits may have been derived from internally generated rhythmic cues corresponding to the components of the APA (Fig. 1) or relative increases in ML-WS duration, although the latter is a likely consequence of increased ML-WS amplitude. We suggest that the observed improvements to gait initiation may represent a combination of the above, driven largely by efficiencies associated with allocating attention specifically towards the production of an APA.

Several studies have contextualised abnormal preparatory movements ahead of attempted gait initiation as representing failed attempts to generate [53] and/or inhibit [20, 54] APA-specific motor programmes. Carlsen et al. [55] suggested that when a motor task (such as gait initiation) is known in advance, cortical demands associated with movement planning and execution can be reduced via the discharge and subsequent inhibition of any relevant motor program to a so-called ‘holding area’. This stored motor program is then subject to self-generated or externally triggered release. Dividing attention may therefore compromise this tonic inhibition, thus releasing APAs that are spatially and/or temporally defective. The notion that defective inhibition of APAs may influence freezing is supported by reports that dual-task paradigms can exacerbate FoG symptoms [56]. As such, external sensory cueing aimed at facilitating gait initiation may be problematic if attending to the cue (and processing the information presented) carries attentional demands that are not directly associated with the most relevant aspects of the ‘primed’ motor programmes.

Many studies have attempted to utilise sensory cues to facilitate gait initiation in people with FoG. However, such strategies have typically specified information regarding the ultimate goal of initiating movement in the stepping limb or completing the initiated step in a given manner (e.g., placing the foot on/over a visual target [5], or imitating the sound [45] or mental image of a stepping action [57, 58]. These attempts to focus attention towards step-related movement goals are reflected in 26/35 of our participants’ self-reporting attempts to consciously initiate foot movement at Baseline (e.g., “pick your foot up”, “put your heel down first”). Therefore, the transition between Baseline to Verbal trials was not characterised by the adoption of conscious strategies per se, but rather a change in the *allocation* of attention between aspects of motor performance (i.e., from the production of a resultant step to an integrated weight-shift and step action) that was sufficient to induce observed improvements (Fig. 6).

It is notable that the relative absence of attentional focus on the resultant successful forward step (in Verbal and Analogy conditions, see Table 2) is indicative of this action maintaining a degree of automaticity; perhaps being triggered by the preceding augmented APA as part of a ‘domino effect’. In this sense, attentional focus on intended foot (rather than APA) movement reported at Baseline might represent attempts to affect the final component of this chain-reaction, resulting in a higher proportion of failed attempts (Fig. 6).

We propose that the newly adopted attentional focus specifically towards APA production may have avoided the need for participants to generate, store and inhibit an independent APA motor programme that would likely be susceptible to problems associated with premature and ineffective release. We propose that the generation of suitably scaled APAs were initiated through, and coupled to, specific pre-potent conscious strategies. This process would, in theory, reduce the burden on deficient ‘automatic’ processes [24, 59, 60] and improve cognitive processing efficiency. More specifically, the above suggestion may constitute a reduction in demands placed on any ‘supervisory system’ [61] otherwise employed to create tonic inhibition on APA motor programmes.

In the current study, we predicted that the use of analogies as a strategy to increase ML-WS would deliver greater benefits, by virtue of potential reductions in attentional demands, compared to the Verbal condition. We observed no evidence of this. Despite FoG-F ML-WS being relatively smaller in Baseline trials (Fig. 5), participants (stepping from a freeze) were able to achieve equivalent ML-WS amplitudes, and successfully initiate forward steps (approx. 100%), during both Verbal and Analogy conditions (Fig. 6). Indeed, the magnitude of improvement in the ratio of successful/unsuccessful steps makes it difficult to make direct comparisons between Verbal and Analogy conditions. Typically, the virtues of implicit approaches to motor learning are evident when comparative verbal processes are complex and vulnerable to breaking down under anxiety. As such, it is possible that verbal strategies used in the current study to initiate gait were sufficiently simple to avoid such issues. After all, descriptions of the APA during training were accompanied by uncomplicated verbal cues that participants were encouraged to use during attempted step initiation (e.g., counting “One, Two, Three” to mark each phase of the APA). Given the weight of evidence from other contexts [30], we maintain that motor performance would show deficiencies if more complex verbal processes were evident, particularly during anxiogenic tasks.

In contrast to the simple verbal cues described above, one might consider the APA-relevant analogies as being relatively complex, and therefore potentially imparting greater demands on cognitive resources relative to the requirements of the task. It is possible that any negative consequences of these increased demands may have been mitigated by the benefits of having internally generated a rich dynamic template to which participants could adhere their movement. In the current study, parity in outcomes observed between Verbal and Analogy conditions suggests that benefits to ML-WS and successful step initiation do not exclusively rely on the explicit conscious control of APA mechanics. This demonstrates clear potential for researchers and clinicians to develop creative solutions for specifying spatial and temporal APA characteristics that are tailored to individual patients’ preferences and requirements. To this end, it should be noted that, in the specific context of APA and step initiation, external sensory cueing strategies carry the following limitation.

Coupling one’s movement to an external source of spatiotemporal information is typically most successful during cyclical and continuous tasks (e.g., finger tapping or walking) as it allows the performer to use estimates of previous errors to adjust and improve subsequent actions. An APA and subsequent step initiation represents a discrete and complex task that cannot be readily adapted and made continuous (and therefore more amenable to external cueing). Therefore, aside from potential logistical issues associated with organising external devices to deliver sensory cues, we propose that internally-generated verbal or imagery cues (such as those evaluated here), represent the most practical solution for people attempting to overcome FoG in daily life. The significant limitation to this suggestion relates to cognitive deficits that have been (albeit inconsistently) associated with FoG (see Gilat and Colleagues [4] for review).

The current study did not attempt to document freezing ‘sub-types’ or categorise participants accordingly [62]. As such, we cannot draw any conclusions regarding the utility of weight-shifting between sub-types or even infer if the results observed are representative of all sub-types. Further work should explore this issue in addition to the potential impacts of specific deficits in cognitive and executive function on the use of the strategies described.

Given the clear improvements observed in the rate of successful gait initiation during both Verbal and Analogy conditions, we suggest that these strategies might represent an effective non-pharmacological option for the practical management of FoG that could be trained in conventional rehabilitation settings. However, given the contrived nature of our stepping tasks and VR environment (including the limitation that participants were required to wear a physical headset), it is important to further evaluate the efficacy and safety of the approaches described in community settings. The current study was also limited by our singular focus on forward walking, meaning that the effects observed cannot be assumed to translate to other variants of attempted gait initiation, such as attempts to turn and change walking direction. Further work is necessary to develop similar strategies to those described here in an attempt to facilitate attempted turns during FoG. We also suggest that future work should aim to record more detailed descriptions of thought processes evident when people attempt to use weight-shifting strategies to overcome FoG in daily life and evaluate possible associations with movement outcomes.

## Conclusions

The current findings illustrate that, when stepping from a freeze, the amplitude of ML-WS is associated with resultant step success. People with FoG pathology were able to voluntarily increase the amplitude of ML-WS (using either Verbal or Analogy strategies) and, when doing so, showed pronounced reductions in the proportion of unsuccessful attempts to initiate stepping from a freeze.

## Data Availability

Data relating to the study described is available at: https://osf.io/5zj47/

## Abbreviations

APA: Anticipatory postural adjustment
COP: Centre of pressure
FES: Falls Efficacy Scale
FoG: Freezing of gait
FOGQ: Freezing of Gait Questionnaire
H&Y: Hoehn and Yahr
ML-WS: Medio-lateral weight-shift
MoCA: Montreal Cognitive Assessment
SIP: Stepping in place
UPDRS-III: Unified Parkinson’s Disease Rating Scale-version III
